# Locally infiltrative inflammatory fibroid polyp of the ileum: report of a case showing transmural proliferation

**DOI:** 10.1093/gastro/gow019

**Published:** 2016-06-09

**Authors:** Shogo Tajima, Kenji Koda

**Affiliations:** 1 Department of Pathology, Shizuoka Saiseikai General Hospital, Shizuoka, Japan; 2 Department of Pathology, Fujieda Municipal General Hospital, Shizuoka, Japan

**Keywords:** inflammatory fibroid polyp, transmural proliferation, subserosa, serosa

## Abstract

Morphologically, an inflammatory fibroid polyp (IFP) is usually centred in the submucosa. Extension of an IFP to the subserosa with destruction of the muscularis propria is exceedingly rare. Herein, we describe a 70-year-old woman who presented with right lower abdominal pain but was finally diagnosed with an IFP. Contrast-enhanced computed tomography revealed a target-like structure with a hypovascular mass at the leading edge, which was consistent with intussusception due to a tumour. Following surgery, the resected specimen displayed a mass measuring 4 × 3 × 3 cm that was protruding into the lumen. Microscopically, the mass was centred in the submucosa, extending up to the mucosal surface and down to the subserosa and serosa. The muscularis mucosae and muscularis propria were destroyed focally. A *PDGFRA* gene mutation in exon 2 (1837_1851 del) that was found in this case, as well as a highly infiltrative growth pattern, strongly supported the neoplastic nature of IFP.

## INTRODUCTION

An inflammatory fibroid polyp (IFP), also known as a Vanek’s tumour, was first described by Vanek in 1949 [1]. The designation of the lesion as an IFP was proposed by Helwig *et al.* in 1953 [2]. IFPs are most frequently found in the stomach, but other sites in the gastrointestinal tract are also involved, and they often occur in patients in their fifth to seventh decades of life, with nearly equal gender distribution [3, 4]. Presenting symptoms are somewhat site-related: gastric IFPs are often found incidentally; small intestinal IFPs are usually associated with intussusception and gastrointestinal IFPs are generally associated with pain and bleeding [5]. IFPs were long considered to be reactive lesions [6]; more recently, however, several studies have revealed that IFPs harbour activating mutations in exons 12, 14, or 18 of the *PDGFRA* gene [7–9]. Although not all IFPs have mutations in *PDGFRA*, they are now postulated to be actual neoplasms [9]; they are also considered benign [3].

Morphologically, an IFP is centred in the submucosa and is composed of bland spindle cells with prominent vasculature; numerous eosinophils are also commonly observed [3]. A unique finding that is sometimes encountered in IFPs is concentric cuffing of vessels by the lesional cells, referred to as 'onion skinning' [3, 10]. Mucosal extension of IFPs through the muscularis mucosae is usually observed [3], but extension outside the muscularis propria, i.e. the subserosa or adventitia, has to date only been reported extremely rarely [11–14].

Herein we present a unusual case of an IFP of the ileum showing extension to the subserosa. The lesion was centred in the submucosa and it destroyed the muscularis propria. The IFP in this case extended as far as the serosa.

## CASE PRESENTATION

**Figure 1.**
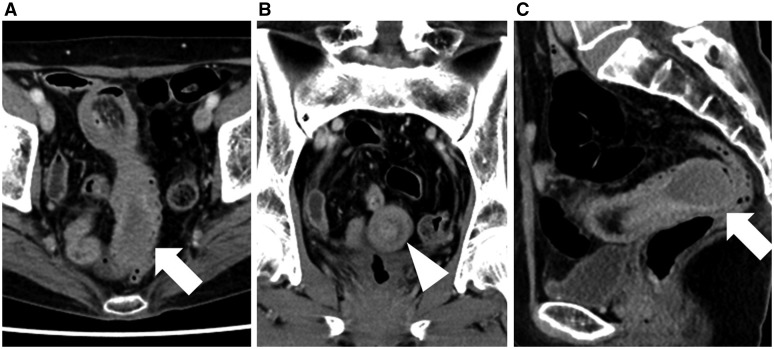
Contrast-enhanced computed tomography. A) Axial; B) Coronal; C) Sagittal images. A target-like structure was revealed with a hypovascular mass (arrows) present at the leading edge. The target-like structure was most evident in (B) (arrowhead).

A 70-year-old woman presented herself at our hospital with right lower abdominal pain. Physical examination revealed slight tenderness in the area; no other remarkable findings were observed. Laboratory tests showed no abnormal results. Following ultrasonography, contrast-enhanced computed tomography was conducted for a suspected intussusception. This revealed a target-like structure with a hypovascular mass present at the leading edge (Figure 1). It was supposed that intussusception had been caused by an unidentified type of tumour; surgery was therefore performed.

**Figure 2.**
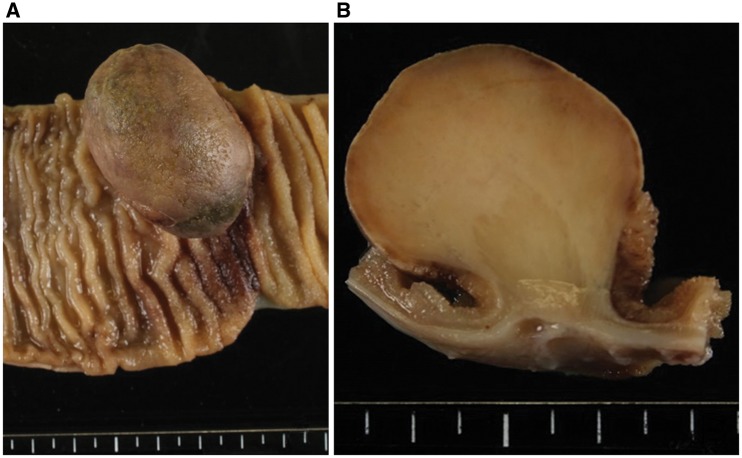
Gross examination. A) A mass was observed protruding into the lumen, with surface ulceration of the ileum. The mass was centred in the submucosa and measured 4 × 3 × 3 cm. B) The cut surface of the mass was yellowish-white with a myxoedematous texture. The lesion infiltrated the muscularis propria with probable subserosal extension.

The surgically resected specimen showed a mass protruding into the lumen, with surface ulceration in the ileum, which was centred in the submucosa and measured 4 × 3 × 3 cm. The cut surface was yellowish white with a myxoedematous texture, and showed infiltration into the muscularis propria, with probable subserosal extension (Figure 2).

**Figure 3.**
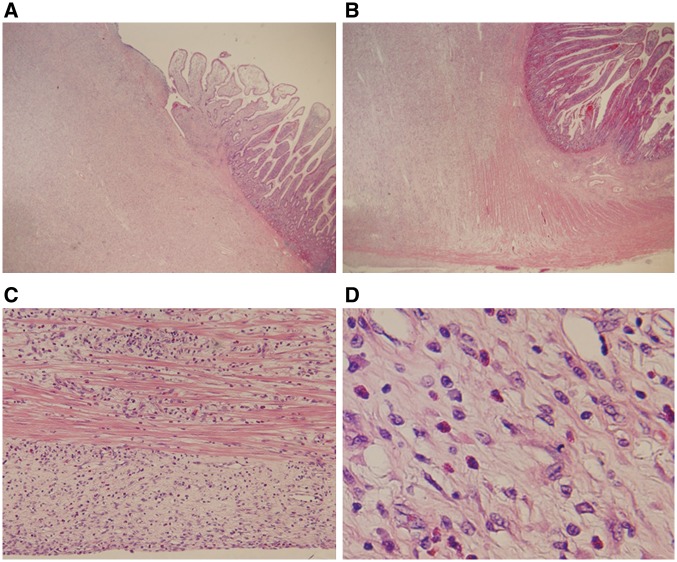
Histopathological findings. A) Extension into the mucosal surface accompanied destruction of the muscularis mucosae and the surface was ulcerated (×20). B) Extension into the subserosa accompanied focal destruction of the muscularis propria (×20). C) The lesion extended as far as the serosa (×100). D) Cellular composition of the lesion was an admixture of fibroblast-like spindled or polygonal mesenchymal cells (not showing atypia) and inflammatory cells, including many eosinophils with modest infiltration of lymphocytes and plasma cells. The background was myxoedematous with tiny collagen fibers and proliferating small- to mid-sized blood vessels (×400).

Histopathology revealed that the lesion was centred in the submucosa and showed transmural growth extending from the mucosal surface to the serosa. This extension into the mucosal surface accompanied destruction of the muscularis mucosae, and the surface was ulcerated (Figure 3A). Extension into the subserosa accompanied focal destruction of the muscularis propria (Figure 3B). The lesion extended as far as the serosa (Figure 3C). Cellular composition of the lesion was an admixture of fibroblast-like spindled or polygonal mesenchymal cells and inflammatory cells, including many eosinophils, with modest infiltration of lymphocytes and plasma cells. No significant atypia and no apparent mitotic figures were observed in the constituent cells; these cells were interspersed in a myxoedematous background with tiny collagen fibres, in which relatively thin and small-to mid-sized blood vessels were proliferating (Figure 3D). So-called 'onion skinning' was not observed.

**Figure 4.**
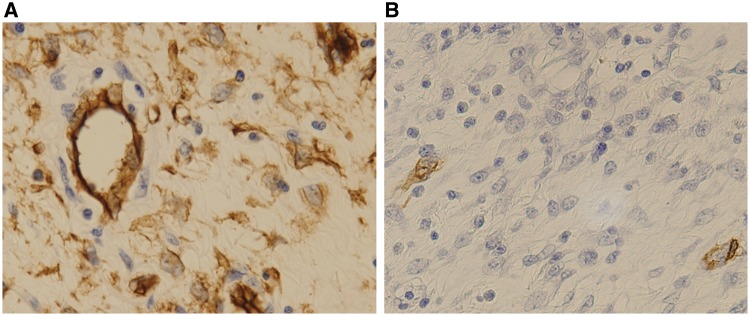
Immunohistochemical findings. A) Lesional cells were positive for CD34 (×400). B) Lesional cells showed focal positivity for alpha smooth muscle actin (×400).

Upon immunohistochemistry (IHC), lesional cells were positive for vimentin (V9, 1:50; Dako, Glostrup, Denmark) and CD34 (QBEnd 10, 1:50; Dako) (Fig. 4A). Focal positivity for alpha smooth muscle actin (αSMA; 1A4, 1:100; Dako) was observed (Fig. 4B). Cells were negative for c-kit (polyclonal, 1:100; Dako), DOG-1 (K9, 1:100, Novocastra Laboratories, Newcastle-upon-Tyne, UK), S100 protein (polyclonal, 1:1000, Dako), and ALK (ALK1, 1:50; Dako). The Ki-67 (MIB-1, 1:100; Dako) labelling index of the lesional cells was less than 1% (data not shown).

A mutational analysis of the *PDGFRA* gene was performed and it was found that deletion was present in exon 2 (1837_1851 del). This analysis was carried out in a commercial laboratory (SRL, Tokyo, Japan). The diagnosis of an IFP was thus confirmed, based on the morphology, the IHC results, and the mutational analysis of *PDGFRA* gene. The patient’s post-operative course was uneventful and she has been recurrence-free for 8 months.

## DISCUSSION

When an IFP presents with typical morphological features such as 'onion skinning' and abundant eosinophils, it is not difficult to diagnose it based on morphology alone. Possible types of lesions included in the differential diagnosis are inflammatory pseudotumours, gastrointestinal stromal tumours (GISTs), and neurogenic tumours [3]. IFPs had previously been linked to inflammatory pseudotumours because of their overlapping features but, following systematic analysis, they are now regarded as distinct entities [15].

A unique feature of IFPs, 'onion skinning' was observed in 54% (45 out of 83) of cases studied) and is thus not always a reliable diagnostic feature [3]. In the absence of 'onion skinning', CD34 expression is a more reliable marker, as it was observed in 100% (12 out of 12) [16] and 86% (38 out of 44) of studied cases [3]; however, both these characteristic features ('onion skinning' and CD34 expression) are sometimes absent, as was shown in 17% (3 out of 18) [17] and 6.8% (3 out of 44) of studied cases [3]. In addition, an atypical IFP that mimicks an inflammatory pseudotumour, lacks 'onion skinning' and CD34 expression, and harbours an activating *PDGFRA* mutation has also been documented [18]. In the present case, although 'onion skinning' was not observed, the presence of many eosinophils, combined with CD34-expressing lesional cells, led to the diagnosis of an IFP.

The transmural proliferation of the IFP with destruction of the muscularis propria extending to the serosa—as seen in this case—is an exceedingly rare observation. IFPs centred in the submucosa only rarely extend beyond the muscularis propria [11–14], and few cases have been observed to infiltrate the muscularis propria [15]. To strengthen the diagnosis of IFP in our case, mutational analysis of *PDGFRA* was performed, because many IFPs are known to harbour activating mutations in exons 12, 14, or 18 of the *PDGFRA* [9]. As was expected, such a mutation was found in our case and it was relatively common one occurring in exon 12 [8].

There are several theories regarding the cellular origin of an IFP. These include a dendritic cell origin [19], a CD34-expressing perivascular cell origin [20], and their arising from 'villous clusters' [8]. The former two are too ubiquitously present around the body to explain the confinement of IFPs to the gastrointestinal tract [14]. Villous clusters are clusters of PDGFRA-expressing mesenchymal cells spreading along the basement membrane of intestinal villi in mouse embryos [21]. It has also been shown that some PDGFRA-expressing cells persist at the same sites in the guts of mice, even into maturity [21]. This theory seems plausible because it reflects the development model of one of the subsets of another type of gastrointestinal mesenchymal tumour, the GIST. In some of them, mutationally activated *PDGFRA* promotes uncontrolled proliferation of precursors of Cajal cells, leading to a neoplasm [8, 9].

In conclusion, this is an extremely rare case of an IFP of the ileum showing transmural proliferation. It was centred in the submucosa and extended to the serosa through the muscularis propria and subserosa with obvious destruction of the muscularis propria. Although 'onion skinning' was not observed, this case is different from an inflammatory pseudotumour, which is normally of a more infiltrative nature than IFP. In differentiating between these two lesions, the presence of many infiltrating eosinophils, along with the expression of CD34, supports the conclusion of an IFP diagnosis.

*Conflict of interest:* none declared.
